# Tribological performance of the biological components of synovial fluid in artificial joint implants

**DOI:** 10.1088/1468-6996/16/4/045002

**Published:** 2015-07-28

**Authors:** Subir Ghosh, Dipankar Choudhury, Taposh Roy, Ali Moradi, H H Masjuki, Belinda Pingguan-Murphy

**Affiliations:** 1 Department of Biomedical Engineering, Faculty of Engineering, University of Malaya, 50603, Kuala Lumpur, Malaysia; 2 Faculty of Mechanical Engineering, Brno University of Technology, Technická 2896/2, 616 69 Brno, Czech Republic; 3 Department of Mechanical and Aerospace Engineering, Monash University, Clayton VIC3800, Australia; 4 Department of Mechanical Engineering, University of Malaya, 50603, Kuala Lumpur, Malaysia

**Keywords:** friction, wear, lubrication, biotribology, biological fluids

## Abstract

The concentration of biological components of synovial fluid (such as albumin, globulin, hyaluronic acid, and lubricin) varies between healthy persons and osteoarthritis (OA) patients. The aim of the present study is to compare the effects of such variation on tribological performance in a simulated hip joint model. The study was carried out experimentally by utilizing a pin-on-disk simulator on ceramic-on-ceramic (CoC) and ceramic-on-polyethylene (CoP) hip joint implants. The experimental results show that both friction and wear of artificial joints fluctuate with the concentration level of biological components. Moreover, the performance also varies between material combinations. Wear debris sizes and shapes produced by ceramic and polyethylene were diverse. We conclude that the biological components of synovial fluid and their concentrations should be considered in order to select an artificial hip joint to best suit that patient.

## 1. Introduction

Artificial hip replacement is one of the most successful achievements in orthopedic surgery. It restores patient mobility and enables a comfortable and independent life [1, 2]. Despite its high success rate, recent data shows that the revision rate is still unexpectedly high; for example, the National Joint Registry of England and Wales reported that overall, 11 and 12% of all total hip replacements failed in 2011 and 2012, respectively [3]. Interestingly, there are a number of research and development (R&D) companies working towards long-lasting artificial joints along with major research universities around the world. Most of their *in vitro* outcomes reveal excellent tribological data, which is contrary to *in vivo* revision rate statistics, thus casting the suitability of the test procedures into question. Notably, the performance of artificial joints does not only depend on implant-related factors such as material and design, but also patient-related factors such as body weight, lifestyle, and synovial fluid; variables which are at times poorly understood or incorporated into *in vitro* testing.

Synovial fluid is considered to be the best lubricant for a natural hip or knee joint. It has unique lubricant properties that protect cartilage and bone surfaces from extremely high contact pressures during most of the patient’s life span. There are four major biological components that make synovial fluid an efficient lubricant, i.e. hyaluronic acid (HA), albumin, mucinous glycoproteins (mainly lubricin), and globulin [4]. All these components were found to have different influences on the outcomes of tests involving synovial fluid. For example, hyaluronic acid helps in increasing the viscosity of synovial fluid [5, 6] at a low shear rate; albumin protects the joint from wearing of articular cartilage [7, 8]; lubricin reduces the shear strength at the asperity contact interface in synovial joints [9, 10] and globulin also plays an important role in the boundary lubrication regime [11]. However, research reveals that synovial fluid composition varies from a healthy person to an osteoarthritis (OA) patient. Healthy knee joints contain the following: albumin (56.6%), globulin (33%), HA (9.4%) and lubricin (1%); whereas OA knee joints contain the following: albumin (56%), globulin (40.4%), HA (3%) and lubricin (0.6%) [6, 12, 13].

The methodology of tribology (*in vitro*) has improved significantly in its ability to replicate an artificial joint in terms of dynamic loadings, multidirectional sliding and temperature. The measured outcomes such as friction, wear, wear debris, and film thickness provide clear evidence of the tribological mechanism. Despite good performance of artificial joints *in vitro*, they are often found to fail *in vivo* after 10–15 years of implantation [14]. To understand this better, some research has emphasized the effect of biological components of synovial fluid on the implanted joints. For example, Myant *et al* [15] provided evidence that film thickness varies with changing fluid content concentration in metal-on-metal hip joints; however, they focused on only two major biological components, i.e. albumin and globulin. Recently, Vrbka *et al* [16] demonstrated (*in situ*) that a hydrophobic surface is more likely to absorb proteins (albumin and globulin); however, they also reported no significant different of metal or ceramic ball on glass disc. Their earlier study confirmed a variation of film thickness of bovine serum with different rolling and sliding conditions. Gispert *et al* [17] also conducted an experiment using a combination of hydrophobic-on-hydrophilic interfaces which revealed that a decreased in friction coefficient with bovine serum albumin (BSA) added to Hank’s balanced salt solution (HBSS) compared to HBSS only. According to McKellop *et al* [18], a HA-added serum shifts the interface lubrication mechanism closer to hydrodynamic from the boundary. Indeed, the lowest friction coefficient was found with ultra-high molecular weight polyethylene (UHMWPE) on cobalt-chromium-molybdenum (CoCrMo) alloy with HBSS+BSA+HA [17]. A polymeric film transfer was observed in these material combinations in the absence of BSA, which caused an increase in wear rate. However, the film transfer stopped when the metallic surface was in contact with added albumin. Since albumin is a soft protein, it is denatured easily with a little rise in temperature. The denatured albumin forms a less stable adsorbed layer on considerably more hydrophilic (alumina) surfaces [17]. As a result, although albumin was added, the lumpy film transfer was not stopped in case of alumina. Further, polymeric film transfer was much more intense when protein was added to the lubricant. Conversely, when two AISI stainless steel and CoCrMo alloys are used with UHMWPE, the wear mechanism and the lumpy film transfer are the same without BSA in the solution. When BSA is added to the solutions, no transfer of polymeric film is observed [17]. As a result, the friction coefficient is found to be low and stable over time. In this case, a stable adsorbed layer reduces the interaction between the solid surfaces, which provides lower lubricated friction [19]. Thus, all of the described studies show an influential tribological behavior of the biological components of synovial fluid. However, no study has been conducted comparing the full ranges of synovial biological components (albumin, globulin, hyaluronic acid and lubricin at appropriate concentrations) that represent an OA patient’s synovial fluid. Our systematic search of tribological behaviors of biological components of synovial fluid [6] also pointed out that the number of studies in this area is comparatively low, and therefore, more research should be conducted in this area. Our recent study [20] on advanced interfaces revealed the better tribological performances under OA-oriented synovial fluid compared to bovine serum. This study focused on modification of surface properties where titanium alloy was used as a bulk material. However, the main complexity of the study was a material transfer from diamond-like carbon, thus, it is really hard to understand the exact role of proteins and HA. Moreover, the tribological performances of individual biological components of synovial fluid along with the full ranges of OA-oriented synovial fluid are yet to be tested to understand their roles in lubrication mechanism. Thus the present study focuses on more practical material combinations such as ceramic-on-ceramic (CoC) or ceramic-on-polyethylene (CoP). Ceramic is more reliable as a head material in hip joints due to its excellent bio-tribological properties that offer low wear and friction rate as well as high scratch resistance, whereas UHMWPE is a good cup material due to its better interaction with body-oriented lubricants like synovial fluid.

Therefore, the objective of this study is to understand the tribological behavior with OA patient synovial fluid in the two best-potential hip prosthesis material combinations, i.e. CoC and CoP. This is the first study that has considered major biological compositions and concentration of OA-affected synovial fluid and their tribological role on artificial joint implants. A total of six types of lubricants (details in table 1) were investigated and prior to the experiment, the physical properties of these lubricants and surface properties of the specimen were measured carefully.

**Table 1.** Composition, viscosity and pH value at 25 °C of the lubricants.

Lubricant	Albumin (mg ml^−1^)	Globulin (mg ml^−1^)	Mucin (mg ml^−1^)	HA (mg ml^−1^)	Other	Viscosity (cP)	pH value
Lubricant 01	—	—	—	—	water	1.14	7.5
Lubricant 02	—	—	—	—	BSF	3.44	7.44
Lubricant 03	31.2	—	—	—	—	1.17	7.20
Lubricant 04	—	31.2	—	—	—	1.17	7.22
Lubricant 05	18	13.1	0.2	—	—	1.35	7.10
Lubricant 06	18	13.1	0.2	1.5	—	1.31	7.22

## 2. Materials and methods

### 2.1. Materials

Bovine metacarpal joints were bought from the local slaughterhouse and exposed for collecting synovial fluid. HA (MP Biomedicals, USA # 0215993350), bovine albumin (MP Biomedicals, USA # 0332), bovine *γ*-globulin (Sigma, US # G5009), and mucin from porcine stomach (type III) (Sigma, US # M1778) were commercially obtained. Mucin (type III), which is similar to lubricin, was used instead of lubricin as it is difficult to extract lubricin from bovine synovial fluid (BSF) due to the many steps of the purification process. The powder-form protein and other components of synovial fluid (HA) were dissolved in phosphate buffered saline (PBS) (Sigma-Aldrich # 4417) at a range of concentrations [12, 13]. The six types of lubricants are described in table 1 along with their physical properties, such as viscosities and pH values, which were measured at 25 °C.

Alumina (99.6% Al2O3) disks and rods were purchased from AdValue Technology, Tucson, USA. To match the dimensions of the tribometer, disks and rods were prepared to a height of 6 mm and diameters of 15 and 6.35 mm, respectively. UHMWPE (Good Fellow, Cambridge, UK) samples were prepared in the same way. The Al_2_O_3_ was polished in several steps. In the first stage, a diamond grinding disc (30 *μ*m) was used for initial polishing and then consecutively followed by 9, 6, 1 and 0.05 *μ*m diamond polycrystalline suspensions (DPS). Surface polishing procedures were not applied to the rods. The 6 × 6.35 mm rods will be hereafter called pins. A dynamic ultra-micro-hardness tester (Shimadzu DUH-211/DUH-211s) was applied in order to measure the modulus of elasticity and hardness of material, and a surface profilometer (Mitutoyo sj210, USA) was used to measure material surface roughness, Ra. The measurement was repeated 5 times at random areas on each sample. The dimensions, hardness, elasticity and roughness of solid materials are shown in table 2.

**Table 2.** The dimensions, hardness, elasticity and roughness of solid materials used.

Samples	Dimensions	Hardness	Elasticity	Roughness (*μ*m)
UHMWPE (pin)	D = 6.35 mm, L = 6 mm	20 MPa	690 MPa	2.0 ± 0.5
Al_2_O_3_ (pin)	D = 6.35 mm, L = 6 mm	5.2 GPa	370 GPa	1.5 ± 0.2
Al_2_O_3_ (disk)	L = 15 mm, W = 15 mm, H = 6 mm	5.2 GPa.	370 GPa	0.15 ± 0.05

### 2.2. Methods

#### 2.2.1. Tribological tests

**Figure 1.**
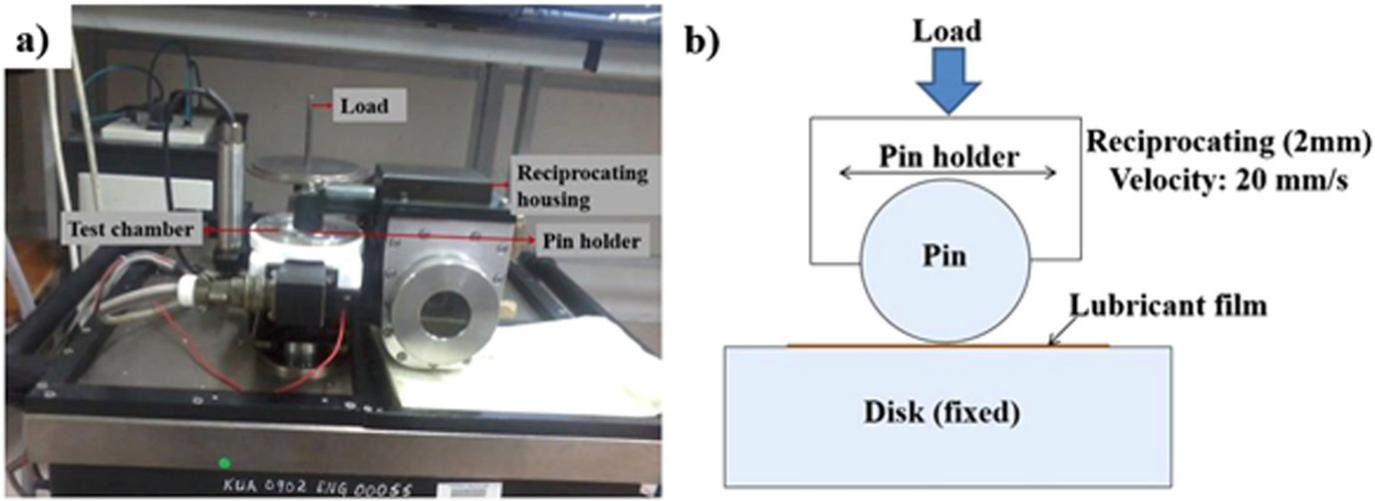
(a) Image of tribometer and (b) schematic of the experimental setup.

A reciprocating pin-on-disk friction tester (figure 1; TR 283 Series, DUCOM, Bangalore, India) was used in the experiment in which contact pressure, speed (20 mm s^−1^) and temperature (37 °C) were maintained in order to simulate hip joint conditions.

The friction coefficients were measured at individual contact pressures (refer to table 3). Total running time for each loading condition was 180 min under each of the applied loads. A pin-on-disk experiment was conducted for friction testing because it is able to provide friction coefficient data with per second resolution. It is worth noting that whilst modern hip simulators are able to vary operating parameters (including dynamic loading, multidirectional sliding directions, and controlled temperature), along with offering a long run capacity, very few of them provide in-run friction coefficient data [21]. Thus, we have used a pin-on-disk tribometer in order to replicate the hip joint in terms of contact pressure, speed, and realistic components of synovial fluid as lubricants. In the current literature, almost all of the frictional or film formation data were collected in the pin-on-disk or ball-on-disk arrangement [16, 22]. The experimental parameters are shown in table 3.

**Table 3.** Experimental parameters.

	Items	Description
	Pin size	Diameter: 6.35 mm, height: 6 mm
	Disk size	Diameter: 15 mm, height: 6 mm
	Speed	20 mm s^−1^
Hertz	Al_2_O_3_/Al_2_O_3_	180, 221, 255 MPa
pressure	Al_2_O_3_/UHMWPE	12, 15, 17 MPa
	Temperature	37 °C

#### 2.2.2. Physical properties of lubricant

The viscosity of the lubricants (10 ml/sample; before and after tests) was measured by using a Brookfield Viscometer –LV (DV-11 + Pro EXTRA) at a 25 °C temperature at definite shear rate 100 s^−1^. The pH meter (AB15 Fisher Scientific Ltd) was used to measure the pH value of the lubricants. The reading was only taken once a stable value was monitored.

#### 2.2.3. Lubricant wettability behavior

The measurement of the static contact angles was carried out through the sessile drop method. The drops were generated with a micrometric syringe and deposited on the substrate surface at 25 °C temperature. A contact angle analyzer (OCA15EC, Data Physics Instruments, Germany) was used to measure these contact angles. The measurements were conducted both before and after the tests in order to find any change in the wettability of the specific substrate.

#### 2.2.4. Wear

##### 2.2.4.1. Surface morphology observation

The surfaces of Al_2_O_3_ disks and rods and UHMWPE rods were examined by field emission scanning electron microscopy (FESEM; AURIGA, Zeiss, Singapore) before and after the tribology test. Through these images, any friction mark produced on the sample surface can be evaluated. If any wear was found, the debris was collected and assessed with FESEM.

##### 2.2.4.2. Wear debris analysis

A few drops of the post-experiment lubricant were collected in order to inspect the morphology of the wear debris. At first, the solution was homogenized in an ultrasonic bath. A few drops of the solution were dried on a glass plate for 24 h at room temperature prior to SEM observation. Energy dispersive x-ray spectroscopy (EDS in SEM; Philips XL40) measurements were carried out in parallel by selecting a rectangular area of wear debris to analyze the chemical composition of the whole area.

##### 2.2.4.3. Wear rate analysis

Wear rate was calculated by measuring weight loss of the disk after the tests. An ultrasonic cleaning was performed to wash out the generated wear debris. Furthermore, the specimens were dried to make sure that there was no weight gain due to lubricant contamination. A digital scale (Oertling VA304) was used for the measurement, which can measure a weight up to 0.010 ± 0.005 mg. Finally, SEM analysis was carried out to see possible wear tracks.

#### 2.2.5. Statistical analysis

IBM SPSS statistics 21 software was used to perform statistical analysis to determine whether there is any significant difference in friction coefficient value between the applied lubricants. A two-way analysis of variance (ANOVA) was conducted on all subsets of data in each study to compare between six types of lubricant over time.

## 3. Results and discussion

### 3.1. Tribological tests

#### 3.1.1. The effect of lubricants on friction coefficient for CoC pair and CoP pair

**Figure 2.**
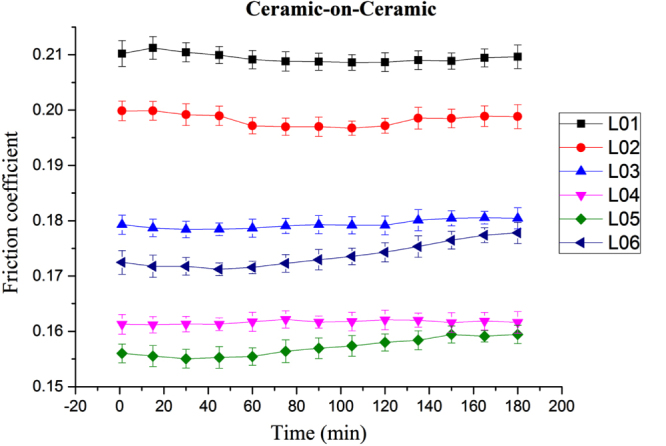
Ceramic-on-ceramic pair friction coefficient profiles over time at 221 MPa (L01, L02, L03, L04, L05, L06 refer to table 1).

The friction profiles over time for various lubricants on ceramic-on-ceramic pairs are shown in figure 2; the effect of different lubricants can be clearly distinguished. L05 (albumin+globulin+mucin) provides the lowest (0.155) and L01 (water) displayed the highest (0.21) friction coefficient. L05 reduced the friction coefficient significantly (26% compared to L02). This results may be attributed to the relative adsorption behavior of protein components of lubricant L05. Moreover, biological component containing all lubricants exhibit a lower friction coefficient profile than that of L01. Our recent study [23] of the lubricating ability of albumin and globulin also demonstrated that albumin and globulin could provide a soft adherent layer between contact interfaces and thus reduce friction. The addition of mucin with albumin and globumin in L05 helps in stable boundary film formation at the asperity contact interface. As a result, the friction coefficient value is minimum for L05. Statistically, there is a significant difference (*p* < 0.05) in friction coefficient value between lubricant types and over time.

Albumin is reported to result in a higher friction coefficient compared to albumin +HA in CoP and metal-on-polyethylene (MoP) prosthesis materials [17]. Globulin yields thicker films, but the films disappear after a certain time and at higher load [15, 16]. In this case, lubricin (mucin) can play a role, along with albumin and globulin [24]. An identical phenomenon was found in the present experiment: at a higher load, the lubricin-oriented lubricant (L05) was one of the best lubricants in terms of friction coefficient reduction in CoC (figure 4).

**Figure 3.**
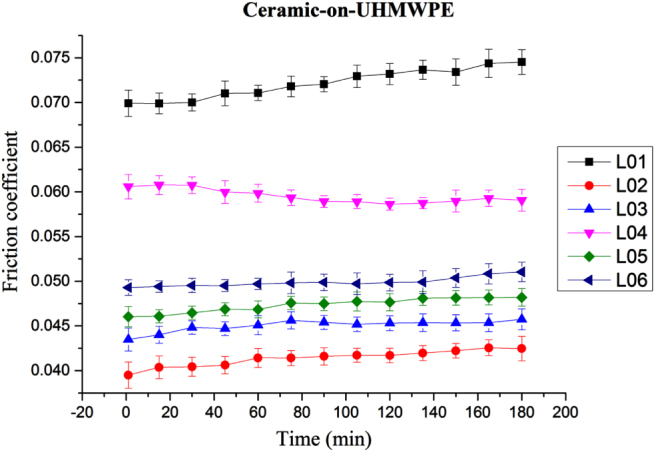
Ceramic-on-UHMWPE pair friction coefficient profiles over time at 15 MPa (L01, L02, L03, L04, L05, L06 refer to table 1).

The friction profiles over time for various lubricants on ceramic-on-UHMWPE pairs are shown in figure 3; L02 (BSF) exhibits the lowest (0.042) and L01 displayed the highest (0.072) friction coefficients. The trends of the friction coefficients differ between the two different material combinations. However, at one point, the phenomenon is similar regardless of any lubricants excluding L01, i.e. the friction coefficient profiles are stable over time. The friction profile of L01 increases significantly over time. There is also a significant difference in friction coefficient value between the six types of lubricant over time.

In CoP, 42% less friction coefficient is achieved by L02 (compared to L01) while L05 exhibited a 35% reduction in friction coefficient. This confirms that the rate of friction coefficient decreases more in the case of CoP than CoC. Both CoC and CoP were found to be sensitive to lubricant biological components during testing. This happened because the friction coefficient is dependent on the deposition rate and consistency of the lubricant on the substrate properties such as wettability and surface tension which influence the deposition nature of the substrate. For example, UHMWPE is comparatively more hydrophobic than ceramic. As a result, the lubrication film on CoP is assumed to be comparatively thicker [16, 25], and the friction coefficient is lower. This result may be attributed to the hydrophobic nature of UHMWPE pin material, which exhibits less attraction to water particles due to its lower surface energy. Hence, proteins can be adsorbed easily onto the hydrophobic surface and produce a stable and comparatively thicker film on CoP than on CoC. However, it is considered a combination of boundary and fluid film formation, yet the lubricant protects the surface and causes higher friction. The stable film formed in the contact region of CoP interfaces enhances lubricating ability and thus lowers the friction coefficient. Thicker films usually lead to higher shear stresses and thus higher friction; however, this happens when the lubrication regime is in hydrodynamic conditions. We believe that the lubrication regime of our experiment is mixed, thus thicker lubrication should lower the frictional coefficient, which happened in our experiment. Since L02 contains almost all combinations of biological components, its effectiveness is more noticeable on the relatively more hydrophobic surfaces such as UHMWPE. On the other hand, water (L01) has no protein or biological component, hence, it is not surprising to find that it produced the highest friction coefficient profile in both CoC and CoP.

Notably, HA (L06) containing lubricant exhibits a comparatively high friction coefficient in CoP and CoC It is worth to mention that HA is responsible to act and perform boundary lubricant, thus, its main function is to protect rubbing surfaces from extreme pressure (as like grease). However, HA has a strong interaction with phospholipid bilayers [26], and hence, it can be more effective in the presence of phospholipids.

#### 3.1.2. The effect of load on CoC pair and CoP pair in the presence of different lubricants

**Figure 4.**
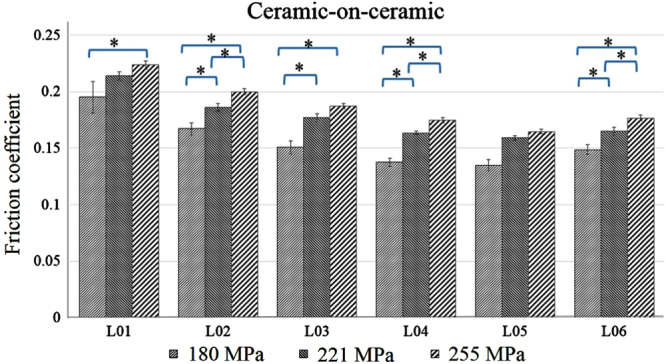
Ceramic-on-ceramic pair friction coefficient profiles at different contact pressure (L01, L02, L03, L04, L05, L06 refer to table 1), ∗ indicates *p* < 0.05 between the load.

**Figure 5.**
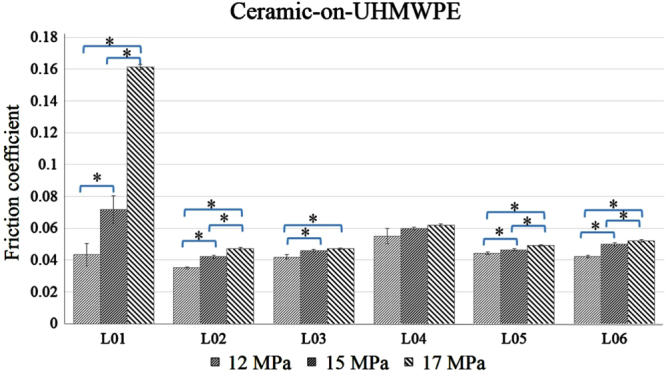
Ceramic-on-UHMWPE pair friction coefficient profiles at different contact pressure (L01, L02, L03, L04, L05, L06 refer to table 1), ∗ indicates *p* < 0.05 between the load.

The effect of changes in load (contact pressure) is presented in figures 4 and 5 for CoC and CoP, respectively. The increased contact pressure escalates the magnitude of the friction coefficient; however, the rate of these changes varies from lubricant to lubricant, and depends on material combination. For example, in the case of CoP, the friction coefficient for water (L01) lubricant increases enormously (0.04 → 0.16) with the increased contact pressure (12 → 17 MPa). On the other hand, friction coefficient profiles for the protein-based lubricants increased almost 5–10% with similar ranges of contact pressure. Interestingly, the increased rate is very similar in CoC, regardless of whether protein or non-protein-based lubricant is used.

At high load, friction force between the contact surfaces increases, providing a high friction coefficient. Although the same load is maintained during the test, the contact pressure between CoC and CoP is different, being comparatively lower for CoP because the hardness and modulus of elasticity of UHMWPE are very low compared to ceramic (table 2). Ching *et al* concluded that the ratio of hardness (H) against modulus of elasticity (E) plays an important role in friction and wear reduction—a high ratio causes lower friction and wear [27]. In our experiment, a similar phenomenon was observed; the ratio for CoP is higher compared to CoC and thus friction coefficient is lower for CoP compared to CoC for all load conditions.

### 3.2. Physical properties of lubricant

#### 3.2.1. Lubricant viscosity

**Figure 6.**
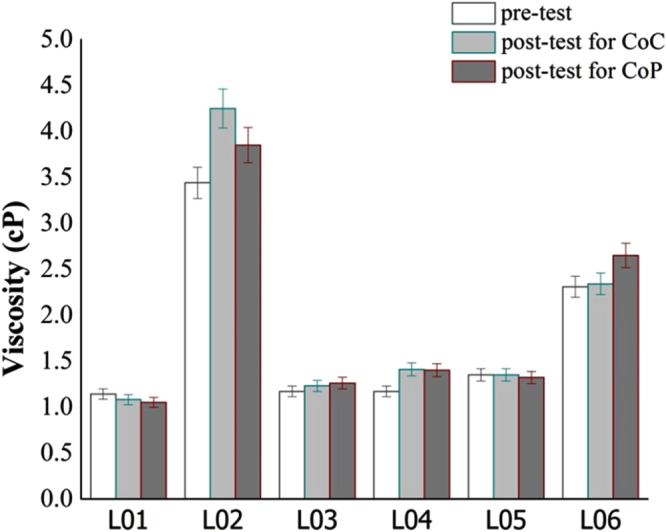
Lubricant viscosities, pre- and post-test conditions (L01, L02, L03, L04, L05, L06 refer to table 1).

The viscosity of each lubricant was measured before and after experiments (figure 6). Most of the lubricants maintained their viscosities, except the viscosity of L02 changed significantly after CoC and CoP tests, and the viscosity of L06 changed after the CoP test.

Lubricant L02 is made up mainly of BSF, which is constituted from many types of protein and lipids, among which HA plays a major role in determining body fluid viscosity [28]. It is not surprising to find that it produced the highest viscosity. However, the mechanisms on how its viscosity increased for the post-tribology test both in CoC and CoP is unclear. But, what is shown here is that the wear debris produced by friction in the contacting area mix with the lubricant to cause a higher viscosity reading. The high viscosity present in L02 lubricants (except water) in choice experiments probably contributes to the high friction coefficient measured (refer to figure 6). In CoP, the process was very reversible, where L02 exhibited the lowest friction coefficient. Therefore, we conclude that the lubricant viscosity properties depend on the tribological contact between different material combinations.

#### 3.2.2. Lubricant pH

Previously it was reported that the pH value of lubricants may have an effect on the lubrication mechanisms [7, 29, 30]. Depending on their pH value, the nature of lubricating properties can be changed depending on their biological component concentrations. In this study, it was found that the difference in pH before and after the tests in all lubricants is less than 1 (refer to table 4).

**Table 4.** The pH value of 6 types of lubricant in three conditions.

			pH value		
Lubricant	Pre-test	Post-test for CoC	Difference	Post-test for CoP	Difference
L01	7.5	7.96	↑0.46	7.99	↑0.49
L02	7.44	7.65	↑0.21	7.59	↓0.15
L03	7.2	7.35	↑0.15	7.68	↑0.48
L04	7.22	7.42	↑0.20	7.65	↑0.43
L05	7.1	7.35	↑0.25	7.45	↑0.35
L06	7.22	7.86	↑0.64	7.59	↑0.37

The small change in value due to deposition of wear debris in the solution does not affect the lubricants’ physical properties. However, the concentrations of biological components in a lubricant greatly affect the pH of the lubricant. Notably, almost all the lubricants were slightly basic at the end of the experiments. Referring to figure 6 and table 4, it is noted that the physical properties of the lubricant before and after test conditions are found to be approximately similar except the viscosity properties of BSF.

### 3.3. Lubricant wettability behavior

Wettability is a key indicator in the relationship between lubricant and surface properties [17]. The wettability of a material is highly dependent on the type of lubricant [31–33]. The different lubricants deposited on the surfaces were determined by the hydrophilic properties of the material. Table 5 presents the contact angle analysis of different lubricants for Al_2_O_3_ and UHMWPE pin materials.

**Table 5.** The contact angle of various lubricants based on pin materials.

	Contact angle (°)					
Materials	*θ*_L01_	*θ*_L02_	*θ*_L03_	*θ*_L04_	*θ*_L05_	*θ*_L06_
UHMWPE (Pin)	86 ± 3	82 ± 3	77 ± 3	74 ± 3	70 ± 3	75 ± 3
Al_2_O_3_ (Pin)	71 ± 3	44 ± 3	50 ± 3	64 ± 3	53 ± 3	48 ± 3

**Figure 7.**
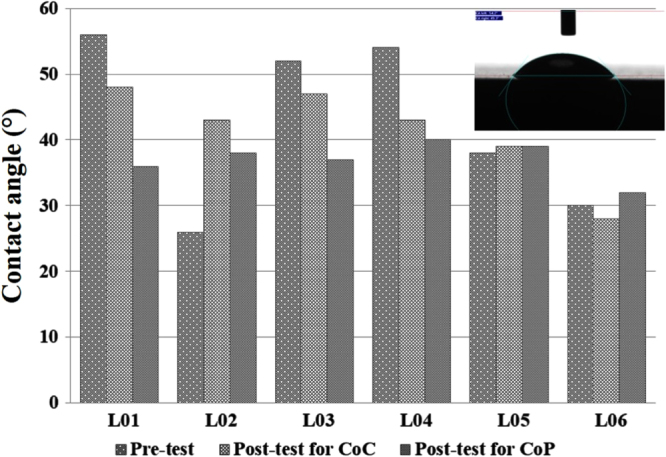
Contact angle of various lubricants on disk surfaces in three different lubricant conditions (pre-test lubricants are the same for CoC and CoP).

Table 5 shows that UHMWPE is more hydrophobic than Al_2_O_3_, which is a finding similar to the previous studies [17]. The surface energy of a material correlates with its contact angle. The higher contact angles of UHMWPE pins reveals the lower surface energy, as well as the hydrophobic nature, of the surface. The lower surface energy reduces the attraction of the surface to water particles, and thus forms a stable layer of lubricant between the contact surfaces. The presence of different lubricants changes the wettability of the material. The contact angles for different lubricant conditions on disk surfaces are presented in figure 7.

The lubricants exhibit different behaviors depending on their interactions with the solid surfaces. L02, which showed the opposite friction coefficient exhibition to CoC and CoP, was also found to have differing wettability properties to Al_2_O_3_ (*θ*_L02_ = 44 ± 3°) and UHMWPE (*θ*_L02_ = 82 ± 3°). With CoC, both pin and disk have a hydrophilic surface by L02, which means higher friction. On the other hand, with CoP, a combination of hydrophilic and hydrophobic surfaces by L02 brings lower friction. Other protein-oriented lubricants (L03–L05) also show slightly different phenomena, i.e. both hydrophilic and similar materials exhibit lower friction (L05), whilst hydrophilic and hydrophobic combination and different materials show a lower friction coefficient. This may be because of the complex behavior of proteins under loading conditions. A stable layer on the surface is formed by protein adsorption on the surface. L02 forms a less stable layer than other lubricants on an Al_2_O_3_ surface in CoC, which can be a reason for a high friction coefficient, resulting in a highly lubricated friction. On the other hand, L02 gives the highest contact angle, compared to other lubricants with UHMWPE pins. When UHMWPE is used as a sliding partner with the Al_2_O_3_ disk, a comparatively more stable layer formed, which exhibits a lower friction coefficient compared to other materials due to a lower lubricated friction between the solid surfaces.

A few studies [31, 33] mention that hydrophilic surfaces are preferable in enhancing tribological outcomes, whilst others [32, 33] have argued that hydrophobic surfaces are preferable for protein deposition. Our tests showed that UHMWPE is more hydrophobic than Al_2_O_3,_ as shown in table 5. The contact angle of each lubricant before and after tests is presented in figure 7. In most cases, the contact angle decreases after testing. This may be caused by the addition of wear debris to the solution during testing. The wear debris was found more in CoP tests. As a result, this wear debris cannot dissolve in the solution, and during measurement of the contact angle it shows interaction with solid surfaces and makes the contact angle lower. Due to trapping of wear particles, it sometimes remains volatile in the solution, thus making the contact angle lower. It was found that L05 and L06 show less variation in the contact angle, which explains the low level of wear debris in the solution, as well as the wear of the material. L06 replicates the synovial fluid of an OA patient, which indicates that the selection of the lubricant composition plays a crucial role in designing artificial hip joints.

### 3.4. Wear

#### 3.4.1. Surface morphology

**Figure 8.**
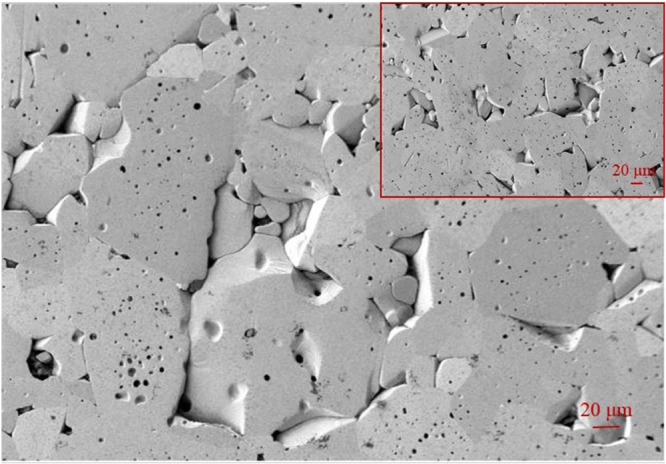
Morphology of Al_2_O_3_ disk surface after friction test. Inset shows SEM image before test.

The surface morphology of the Al_2_O_3_ disk specimen after testing is shown in figure 8, where the SEM image (inset) was taken before the test. Nearly zero wear was found on Al_2_O_3_ disk surfaces. There are some pores observed on Al_2_O_3_ disk surfaces, but these are not considered wear.

**Figure 9.**
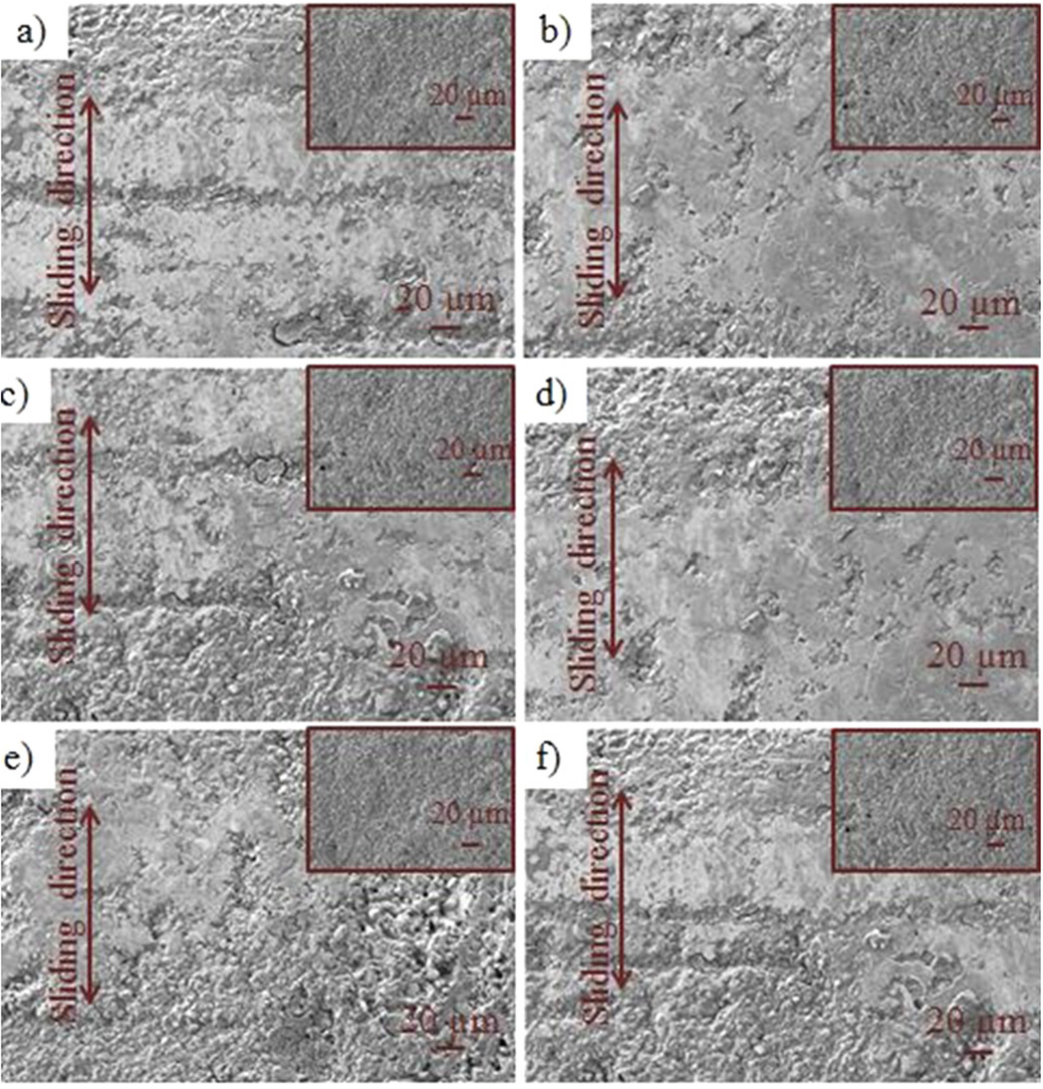
SEM images of Al_2_O_3_ pin after testing in presence of (a) L01, (b) L02, (c) L03, (d) L04, (e) L05), (f) L06 (L01, L02, L03, L04, L05, L06 refer to table 1). Insets show SEM images before tests.

There are some visible wear signs on the Al_2_O_3_ pins (figure 9), especially the Al_2_O_3_ pin tested under L01 (water) which shows more visible wear compared to other surfaces (figure 9(a)). Less wear is observed in the case of lubricants L05 and L06 respectively (figures 9(e) and (f). A lower wear and lower friction coefficient indicate a better capability of L05 in the CoC hip joint prosthesis. When the comparatively rougher alumina pins rubbed against polished alumina disks during friction tests, wear particles were generated by gradual loss of material from rougher surfaces. Hence, the rougher surfaces become smoother in the contact area. Although, both pin and disk are alumina, visible wear signs were only found on pin surfaces because peak valley rougher surfaces faced relatively higher contact pressure due to their lower real contact area.

Notably, HA-containing lubricant L06 had low wear, although it exhibited a high friction coefficient profile. Thus, it could be concluded that HA has better wear resistance properties because it is highly viscous and more hydrophobic on ceramic surfaces, certain physical properties that can reduce the wear rate in the sliding interface. As other studies [32, 34, 35] have also pointed out, viscosity and contact angle of lubricant plays an important role in reducing wear in the contact interface.

**Figure 10.**
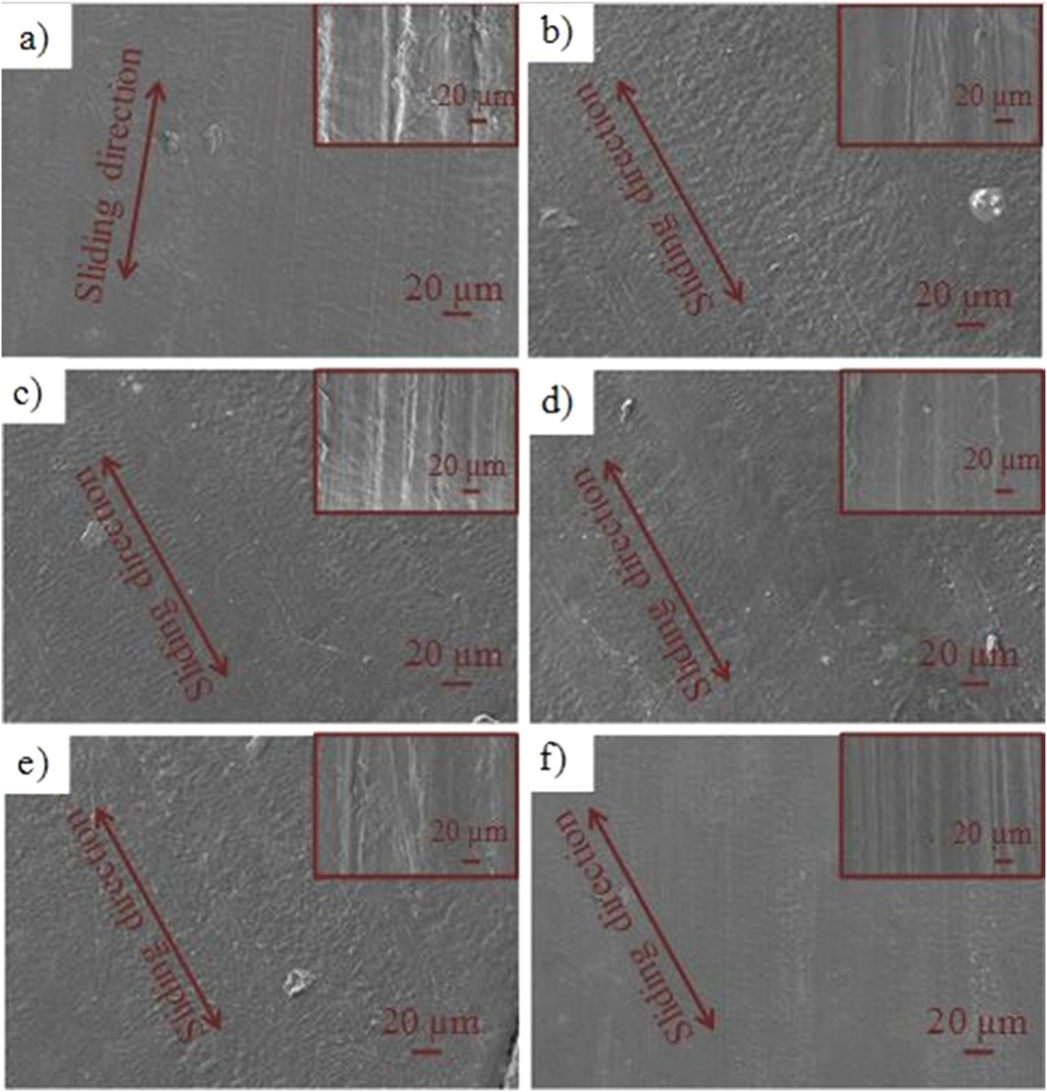
SEM images of the UHMWPE pin after testing in presence of (a) L01, (b) L02, (c) L03, (d) L04, (e) L05), (f) L06 (L01, L02, L03, L04, L05, L06 refer to table 1). Insets show SEM images before tests.

It is worth noting that the wear sign was evaluated based on the smoothness of the surface since the initial pin surfaces were quite rough for the pin material (figure 10). The morphology of worn UHMWPE pin material surfaces after CoP experiments is presented in figure 10. L02 (figure 10(b)) provides fewer worn surfaces on UHMWPE, and this agrees with the friction coefficient profile of CoP; the L06 lubricant, which contains HA, showed more wear in the UHMWPE pin, which is in contrast to the ceramic pin. Therefore, it can be concluded that HA plays a significant role in the lubrication mechanism; however, its performance is material property dependent.

#### 3.4.2. Wear debris analysis

**Figure 11.**
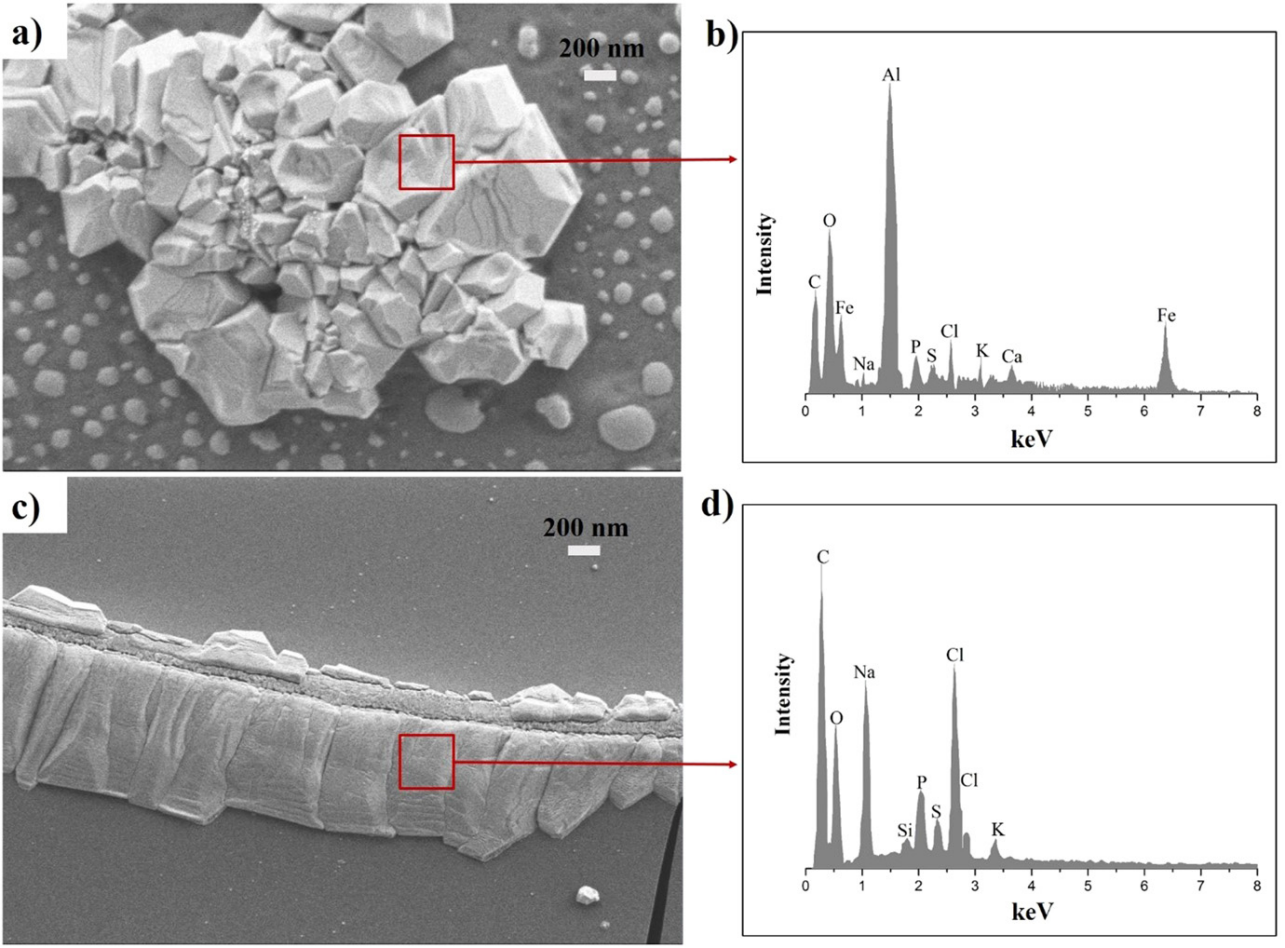
SEM images of wear debris in ceramic (a) and UHMWPE (c); EDS analysis of wear debris in ceramic (b) and UHMWPE (d).

The morphology of wear debris can be a contributor, as can be third body abrasive wear, and they tended to react with a biological response. Thus, a wear debris inspection is very important to predict its role in the contacting region and periprosthetic tissue [36]. The accumulated wear debris nanoparticles of Al_2_O_3_ and UHMWPE materials are presented in figures 11(a) and (c), respectively.

Al_2_O_3_ particles are comparatively smaller than UHMWPE particles. UHMWPE wear debris is thin and long in size. The wear debris found in ceramic is in the 0.4–1.3 *μ*m range, and for UHMWPE is in the 1.2–2.33 *μ*m range, similar to the previous results [36, 37]. Our preliminary wear debris study did not find any significant variation of wear particle size and shape in respect to various lubricants.

EDS allows us to perform qualitative and quantitative chemical analysis of the studied sample [38]. Our EDS results showed the elemental energy spectra of different components, which confirms the presence of Al and O in ceramic, and C in UHMWPE wear debris at high concentration. The other foreign elements (Na, P, S, Cl, K) which are found at low intensity in the EDS report can be explained by the deposition of powder-form protein on wear particles at a lower rate. However, this is a preliminary wear debris analysis study. Further studies can be carried out with detailed chemical analysis for various lubricants.

#### 3.4.3. Wear rate

The reduction in wear rate is desirable for artificial joint implants. The weight of each pin and disk sample was measured before and after the experiment for each lubricant. The wear rate was calculated based on weight loss of the sample after test. Wear rate profiles are as follows: L05 < L04 < L06 < L03 < L02 < L01 for the ceramic pin in CoC and L02 < L05 < L03 < L06 < L04 < L01 for the UHMWPE pin in CoP. The wear rate falls within the range of 0.007–0.02% for the ceramic pin and 0.05–0.17% for the UHMWPE pin. The calculated average wear rates of pin materials for different lubricants are presented in table 6.

**Table 6.** Wear rates of pin materials for various lubricants.

	Ceramic pin weight (g)			UHMWPE pin weight (g)		
Lubricant	Pre-test	Post-test	% of wear	Pre-test	Post-test	% of wear
L01	0.703 94	0.703 74	0.02	0.189 67	0.189 34	0.17
L02	0.684 46	0.684 35	0.01	0.187 46	0.187 36	0.05
L03	0.689 48	0.689 39	0.01	0.187 37	0.187 13	0.12
L04	0.712 37	0.712 27	0.01	0.187 97	0.187 66	0.16
L05	0.726 83	0.726 78	0.007	0.188 81	0.188 63	0.09
L06	0.715 33	0.715 27	0.008	0.185 83	0.185 64	0.10

Overall, the wear rate is directly affected by the friction coefficient produced by each lubricant. Wear rate increases with increasing friction coefficient value both in CoC and CoP over the lubricants (except L05 in CoP). In CoP, L05 provides a lower wear rate compared to L03 although L05 is higher in friction coefficient value than L03, which confirms the better lubricating ability of lubricin-oriented lubricants.

Usually, high friction causes more wear on contact surfaces [20, 39]. However, there are many other factors related to the wear mechanism. An extremely hard material rubbing against a softer material causes more wear on softer surfaces. In our experiment, wear rate is comparatively high for UHMWPE pin materials. Surface roughness played a significant role in wear rate [40] along with biological components of synovial fluid.

## 4. Conclusions

The paper reports a fundamental investigation of the tribological role of synovial fluid compositions in artificial hip joint implants. The key findings of this research are as follows.

(1) The biological components of synovial fluid were found to have individual roles in friction coefficient and wear. L05 and L06 exhibited lower friction in CoC whereas L02 showed lower friction in the case of CoP. However, L01 provided the highest friction coefficient in both cases because of its poor load-withstand ability.
(2) With all load conditions, lubricin-oriented lubricants (L05, L06) provided better tribological performances in CoC and BSF (L02) in CoP by minimizing friction and wear.
(3) The viscosity values for L02 and L06 were found to be higher compared to other lubricants because they contain HA, which tends to increase the viscosity of the lubricant. This higher viscosity value was found to be less effective in the absence of phospholipids to reduce the friction coefficient value. However, the pH values of pre- and post-lubricant were found to be approximately similar.
(4) The higher contact angle for UHMWPE materials indicated its hydrophobic nature, which helps in stable boundary layer formation because surface energy decreases with increased contact angle, thus attracting fewer water particles and facilitating protein adsorption. Moreover, the contact angle values varied from lubricant to lubricant, depending on its components and their interaction with the surfaces.
(5) Material for hip or knee joint prosthesis design should be considered with respect to OA patient synovial fluid composition and concentration because it will reveal the actual physiological condition of the patient.
